# Travel times to hospitals in Australia

**DOI:** 10.1038/s41597-019-0266-4

**Published:** 2019-11-01

**Authors:** Sebastiano Barbieri, Louisa Jorm

**Affiliations:** Centre for Big Data Research in Health, University of New South Wales, Sydney, NSW Australia

**Keywords:** Public health, Health services

## Abstract

Travel time to hospital is a key measure of health service accessibility, and impacts patients’ experiences of care and health outcomes. Methods used to estimate travel time vary across studies. In Australia the smallest geographical areas defined by the Australian Bureau of Statistics for the release of population counts are mesh blocks (MBs) and the smallest geographical areas for the release of health-related statistics are statistical areas level 2 (SA2). SA2s are built up from whole MBs. This project used the Open Source Routing Machine (OSRM) HTTP server to compute estimated travel times between the centroid of each inhabited MB and each hospital in Australia, as well as the shortest travel times between MBs and any hospital. By computing population-weighted averages across MBs, the average travel times to hospitals and the shortest travel time to any hospital were estimated for each SA2. This dataset will promote consistency across studies investigating geographic influences on health care in Australia, and the methods are applicable to generating similar datasets for other countries.

## Abstract (summary)

**Table:** 

Measurement(s)	temporal_interval • Hospital
Technology Type(s)	computational modeling technique • digital curation
Factor Type(s)	geographic location
Sample Characteristic - Location	Australia

Machine-accessible metadata file describing the reported data: 10.6084/m9.figshare.9971222

## Background & Summary

Patients who live farther from healthcare facilities have lower rates of usage after adjustment for need than those who live closer, an effect documented as the *distance decay* association^1,2^. Further, longer travel times to healthcare facilities may be associated with worse health outcomes for patients^3^. This is particularly relevant in low-income countries, where health services are scarce, but also in more developed countries where settlement is spread thinly over vast areas, as in interior parts of Australia or North America^1^.

Geographical access to health services is usually measured using car travel times estimated by a geographic information system (GIS)^4^. The GIS is used to find the shortest travel time from each population location to each healthcare facility along the road network, by taking into account information about road length and average travel speeds along successive segments of road^5^. Travel time estimates are recognized as more appropriate measures of travel effort than straight-line distances, particularly in regions without an extensive road network, with physical barriers such as major rivers or mountains, or with an irregular coastline^6^. However, the methods used to estimate travel time to healthcare facilities vary between published studies. A recent systematic review reported that 19% of studies did not clearly state how they had calculated this variable, while straight-line distance was used in >25% of studies^3^.

Some studies internationally have used geocoded patient address to estimate travel times to healthcare facilities^3^, but to preserve patient confidentiality, exact address information is not generally available to researchers using health data in Australia. Accordingly, average travel times for residents of small geographical areas generally present the most suitable option. Where patient addresses are used, routing engines that can be run locally are preferable to online routing services such as Google Maps^7^, which require uploading of address information.

**Fig. 1:**
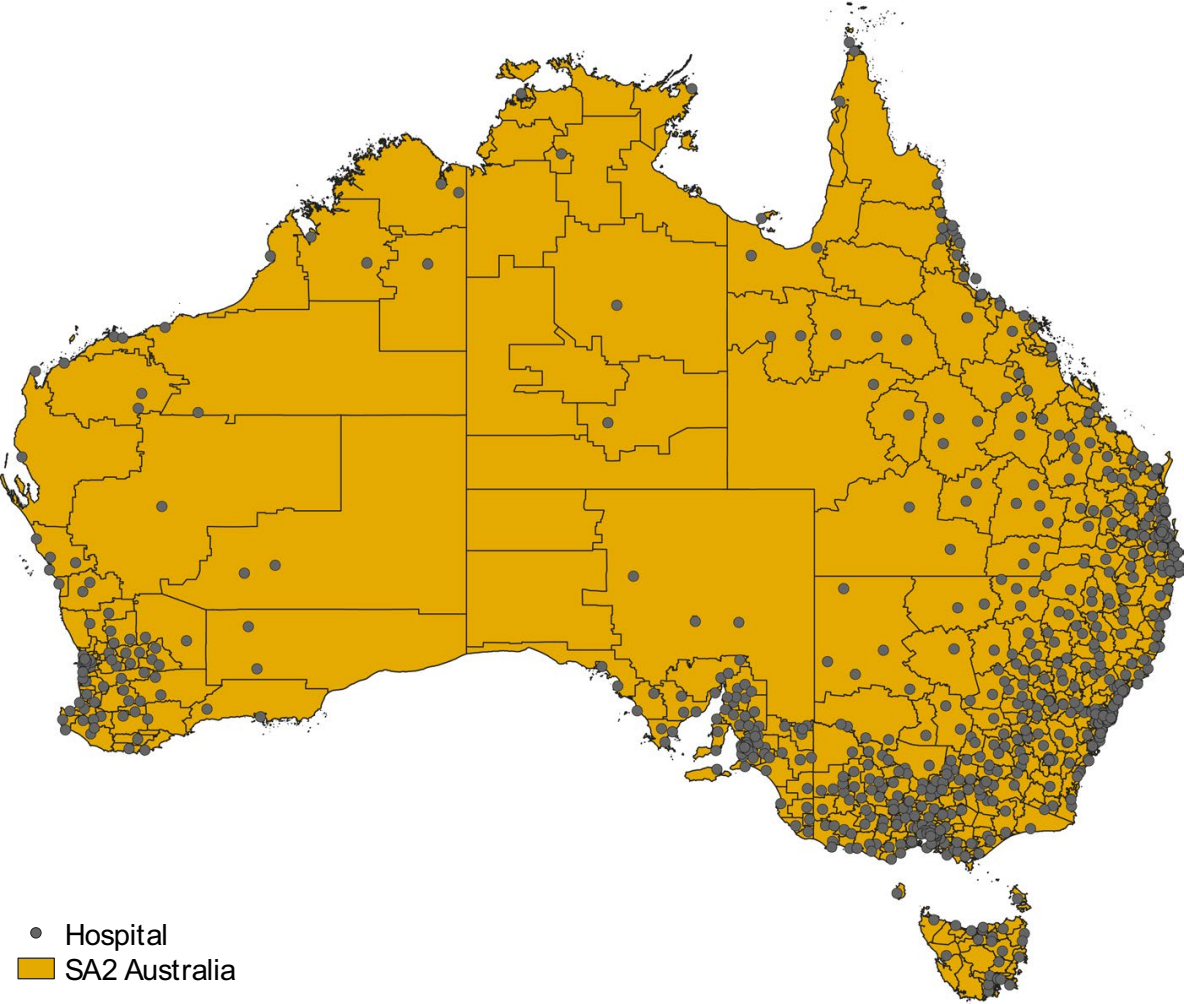
Map of Australia subdivided into statistical areas level 2 (SA2s) and with hospitals represented as grey dots.

The smallest geographical areas for the release of Australian Bureau of Statistics (ABS) non-census and intercensal statistics, including health and vitals data, are the statistical areas level 2 (SA2). The purpose of SA2s is to represent communities that interact together socially and economically. There are 2,310 SA2 regions covering the whole of Australia without gaps or overlaps (including 18 non-spatial SA2 special purpose codes, such as migratory-offshore-shipping and no-usual-address codes for each state and territory)^8^. The smallest geographical areas defined by the ABS are mesh blocks (MB). There are 358,122 mesh blocks covering the whole of Australia (including 113 non-spatial MB special purpose codes); SA2s are built up from whole MBs^9^. Counts of the total usual resident population are available for each MB based on the 2016 Australian census of population and housing^10^. The majority of populated MBs contain between 30 to 60 dwellings^10^; the median surface area of populated MBs is 0.0371 km^2^ (range: 0.0004–165,571 km^2^). Finally, information about the geographic location of 1,011 hospitals in Australia is provided by the Australian Institute of Health and Welfare^11^. Figure 1 shows the location of hospitals in Australia and the subdivision of the country into SA2s.

**Fig. 2:**
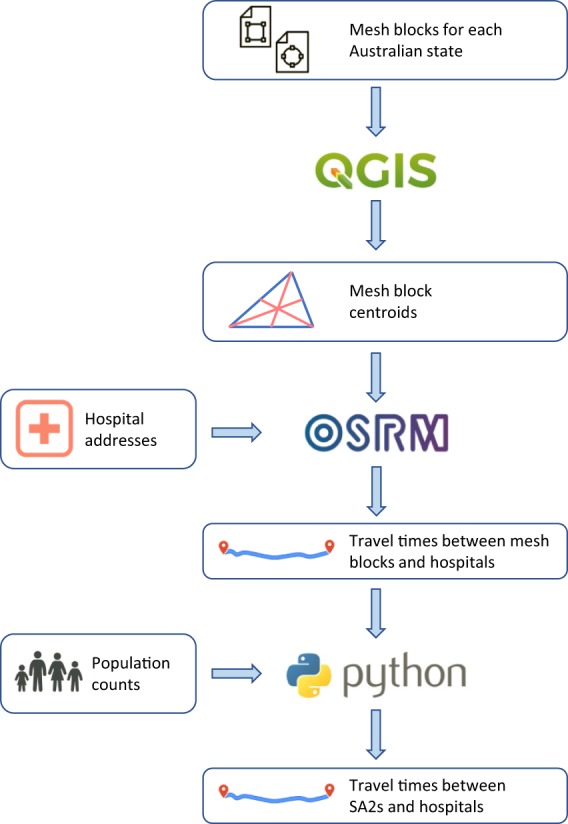
Schematic overview of the process used to compute travel times between mesh blocks and hospitals as well as between statistical areas level 2 (SA2s) and hospitals.

The purpose of this project is to create a table with travel time estimates between each SA2 and each hospital in Australia, as well as a table with the shortest travel time to any hospital, averaged across residents of a SA2. As a by-product of these analyses, tables with travel time estimates between each MB and each hospital, and with shortest travel time from a MB to any hospital were created as well. When used in combination with hospital morbidity data containing patient SA2 of residence, the dataset can be used to derive patient-level variables for analysis, including the distance travelled to hospital and whether the patient travelled to the closest hospital or not, and hospital-level variables, such as the average travel time for patients attending a particular hospital. A schematic overview of the process used to create this dataset is presented in Fig. 2.

This dataset is released into the public domain with the goal of facilitating research on access to healthcare services and health outcomes in Australia, and ultimately to promote geographical equity in healthcare. Its availability will encourage consistency across studies investigating geographic influences on healthcare in Australia and the methods are applicable to generating similar datasets for other countries.

## Methods

### Data quality

The list of hospitals in Australia distributed by the Australian Institute of Health and Welfare on the MyHospitals website contained, as of April 2019, all 690 public hospitals in Australia and 321 private hospitals^11^; however, some private hospitals might have been missing since participation in MyHospitals by private hospitals is voluntary. Further details on data quality assurance are available online^12^. Verification of the geographic coordinates of 10 randomly sampled hospitals in each Australian state and territory (where present) did not indicate any inaccuracies.

OpenStreetMap data for Australia, updated daily, is available for download^13^. Previous work determined that in January 2016, coverage of the Australian road network by OpenStreetMap was 94%^14^. Global coverage increased from 83% in January 2016 to 89% in May 2017^14^; country-specific increases were not reported but coverage of the Australian road network is likely to have improved as well.

### Computation of mesh block centroids

MBs for the different Australian states and territories were downloaded in shapefile format^15^ to a personal computer (Intel Core i7-6600U CPU at 2.60 GHz, 16GB of RAM). The QGIS geographic information system, version 3.6.2, was used to merge these MB files and compute the centroids of all spatial MBs. The MB centroids were exported as a comma-separated values (csv) file and merged with the table of population counts based on MB code.

### Computation of travel times between mesh blocks and hospitals

OpenStreetMap data for Australia^13^ was used to initiate a local instance of the Open Source Routing Machine (OSRM) HTTP server (version 5.16.0 on Windows 10). The table service of the OSRM server was used to query the car travel time of the fastest route between each centroid of spatial, inhabited, MBs and each hospital in Australia. The OSRM server does take into account possible ferry services but may not return a valid travel time if no route is found (e.g. there are currently no passenger ferries to King Island in Tasmania where the King Island District Hospital and Health Centre is located). The OSRM server supports the concurrent processing of multiple requests and the total processing time for creating the full travel time matrix between MBs and hospitals was approximately 10 hours. Given the travel times matrix between all MBs and hospitals it was possible to compute the shortest travel time between each MB and any hospital.

### Computation of Travel Times Between SA2s and Hospitals

Averaging across MBs in each SA2, weighted by population, determined the travel times matrix between SA2s and hospitals, as well as the average shortest travel time to a hospital for residents of a SA2. These steps were performed using Python version 3.6.4.

## Data Records

Data records are stored on figshare^16^ and specific details on file size and format are reported in Table 1.

**Table 1:** Format and size of data records. csv: comma-separated values; pkl: pickle; osm.pbf: OpenStreetMap protocolbuffer binary format; NA: not applicable.

Name	Format	Size [KB]	Num. Rows
mb_2016_centroids	csv	70,520	358,122
myhospitals-contact-details	csv	197	1,011
duration_mb_hospitals	pkl	2,839,815	358,122
duration_mb_hospital_shortest	csv; pkl	17,792; 16,789	358,122
duration_sa2_hospitals	csv; pkl	40,360; 18,283	2,310
duration_sa2_hospital_shortest	csv; pkl	66; 56	2,310
australia-latest	osm.pbf	389,299	NA

The “mb_2016_centroids” table contains the 2016 MB codes (MB_CODE16 column), names and codes of the corresponding SA2 (SA2_NAME16 and SA2_5DIG16), centroid longitude (xcoord) and latitude (ycoord). The “myhospitals-contact-details” table contains the names of hospitals in Australia, their geographic coordinates (Longitude and Latitude) and an assigned hospital identifier between 1 and 1,011 (Hospital_ID). The “duration_mb_hospitals” table contains the 2016 MB codes (MB_CODE16) and the travel time in seconds to each hospital (time_to_X columns, where X is the Hospital_ID). The “duration_mb_hospital_shortest” table contains the 2016 MB codes (MB_CODE16) and the shortest time in seconds (shortest_time_sec) and minutes (shortest_time_min) from the MB’s centroid to any hospital. The “duration_sa2_hospitals” and “duration_sa2_hospital_shortest” tables are analogous to “duration_mb_hospitals” and “duration_mb_hospital_shortest”, respectively, for SA2s instead of MBs. Finally, “australia-latest” contains the OpenStreetMap data that was used for computing travel times to hospitals^13^.

## Technical Validation

For each Australian state and territory, 1,000 routes between MB centroids and the closest hospitals were sampled without replacement (8,000 routes in total). Estimated travel times were compared between OSRM and the Google Maps Distance Matrix service^7^. Note that neither model considered the time that may be required to reach the road network, current traffic conditions, temporary road closures, or modes of transport other than travel by car. Mean differences in seconds (Google Maps time – OSRM time) with corresponding 95% confidence intervals are reported in Table 2. The estimated travel times were comparable, with Google Maps being slightly more conservative. Differences in estimated travel times were, on average, less than 5 minutes. Differences were smaller in Queensland and New South Wales and larger in the Northern Territory and the Australian Capital Territory.

**Table 2:** Average differences in estimated travel times computed by Google Maps and by the Open Source Routing Machine (OSRM) across Australian states and territories.

State or Territory	Mean Travel Time Difference between Google Maps and OSRM [sec] (95% Confidence Interval)
New South Wales	35 (20, 51)
Victoria	54 (44, 64)
Queensland	22 (−6, 50)
South Australia	49 (36, 63)
Western Australia	40 (4, 75)
Tasmania	100 (2, 197)
Northern Territory	138 (6, 269)
Australian Capital Territory	115 (111, 120)
“Overall”	69 (48, 90)

**Fig. 3:**
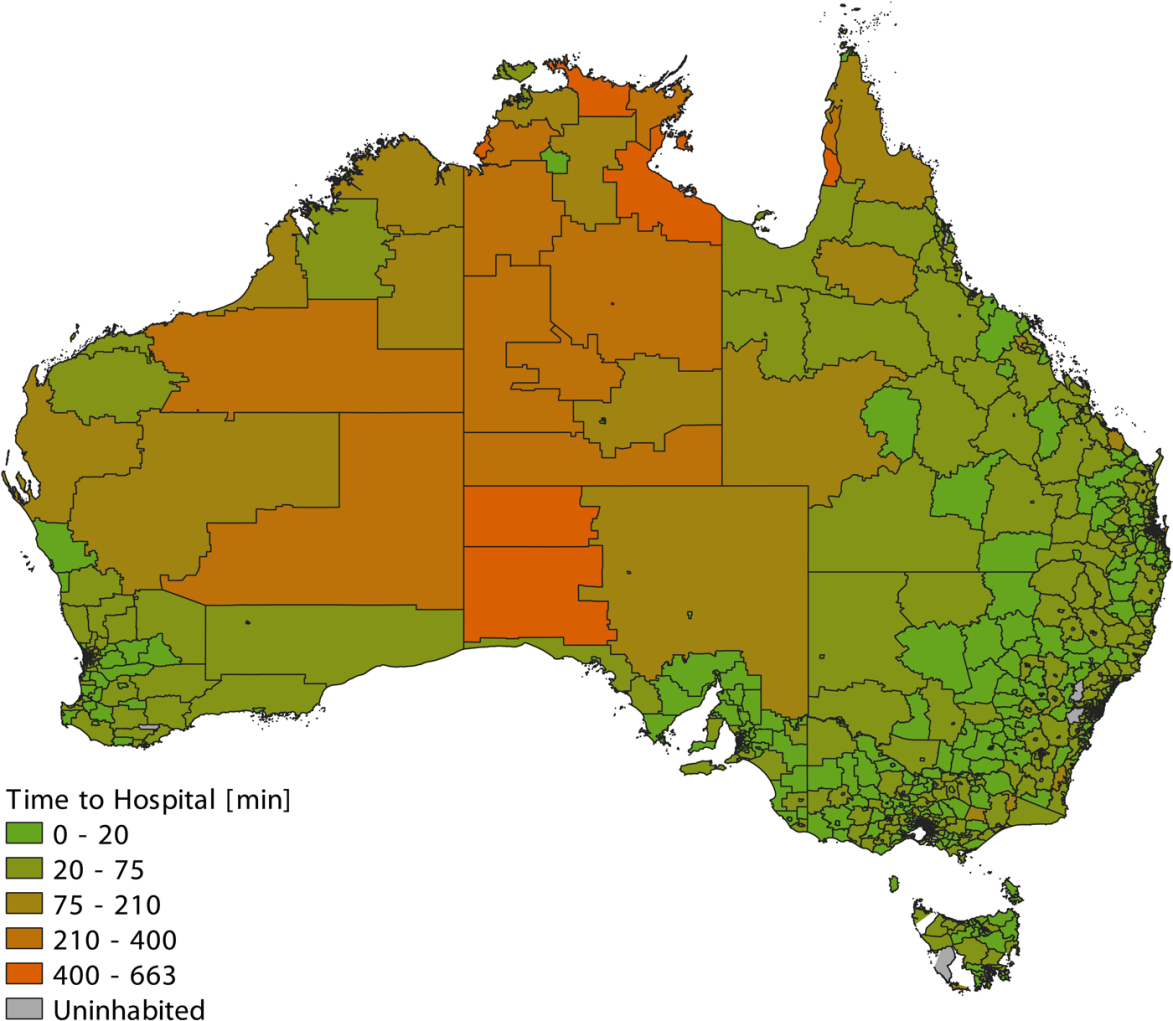
Map of Australia subdivided into statistical areas level 2 (SA2s) and color-coded according to the shortest travel time to a hospital.

**Fig. 4:**
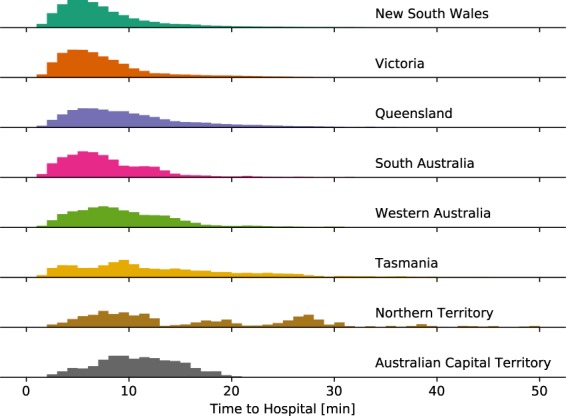
Distribution of shortest travel times to a hospital in Australian states and territories.

Figure 3 shows a map of Australia subdivided into SA2s and color-coded according to the shortest travel time to a hospital. Figure 4 shows histograms of shortest travel times to a hospital, computed based on population-weighted MB data, for each Australian state and territory.

## Data Availability

The Python 3 code related to this project is available for download at https://github.com/sebbarb/times_to_hospitals_AU.
